# Towards a machine‐learning assisted diagnosis of psychiatric disorders and their operationalization in preclinical research: Evidence from studies on addiction‐like behaviour in individual rats

**DOI:** 10.1111/ejn.15839

**Published:** 2022-11-01

**Authors:** Kshitij S. Jadhav, Benjamin Boury Jamot, Veronique Deroche‐Gamonet, David Belin, Benjamin Boutrel

**Affiliations:** ^1^ Center for Psychiatric Neuroscience, Department of Psychiatry Lausanne University Hospital Lausanne Switzerland; ^2^ Cambridge Laboratory for Research on Impulsive/Compulsive spectrum Disorders (CLIC), Department of Psychology University of Cambridge Cambridge UK; ^3^ Université de Bordeaux, INSERM, Neurocentre Magendie Bordeaux France; ^4^ Division of Adolescent and Child Psychiatry, Department of Psychiatry Lausanne University Hospital Lausanne Switzerland

**Keywords:** addiction, clustering, individual vulnerability, machine learning, neural networks, substance use disorder

## Abstract

Over the last few decades, there has been a progressive transition from a categorical to a dimensional approach to psychiatric disorders. Especially in the case of substance use disorders, interest in the individual vulnerability to transition from controlled to compulsive drug taking warrants the development of novel dimension‐based objective stratification tools. Here we drew on a multidimensional preclinical model of addiction, namely the 3‐criteria model, previously developed to identify the neurobehavioural basis of the individual's vulnerability to switch from controlled to compulsive drug taking, to test a machine‐learning assisted classifier objectively to identify individual subjects as vulnerable/resistant to addiction. Datasets from our previous studies on addiction‐like behaviour for cocaine or alcohol were fed into a variety of machine‐learning algorithms to develop a classifier that identifies resilient and vulnerable rats with high precision and reproducibility irrespective of the cohort to which they belong. A classifier based on K‐median or K‐mean‐clustering (for cocaine or alcohol, respectively) followed by artificial neural networks emerged as a highly reliable and accurate tool to predict if a single rat is vulnerable/resilient to addiction. Thus, each rat previously characterized as displaying 0‐criterion (i.e., resilient) or 3‐criteria (i.e., vulnerable) in individual cohorts was correctly labelled by this classifier. The present machine‐learning‐based classifier objectively labels single individuals as resilient or vulnerable to developing addiction‐like behaviour in a multisymptomatic preclinical model of addiction‐like behaviour in rats. This novel dimension‐based classifier increases the heuristic value of these preclinical models while providing proof of principle to deploy similar tools for the future of diagnosis of psychiatric disorders.

## Graphical abstract

The diagnosis of a substance use disorder (SUD) in humans is solely based on the presentation of the individual being clinically assessed. Such individual‐based determination of the presence of an addiction‐like phenotype is lacking even in the most sophisticated preclinical models of SUD. We deployed a clustering/artificial neural network‐based machine learning strategy based on the multidimensional 3‐criterion model of addiction to develop a classifier that systematically and objectively determines if any individual rat with a prolonged history of cocaine or alcohol self‐administration, taken independently from the cohort to which it belongs, is positive or negative for addiction‐like behaviour.

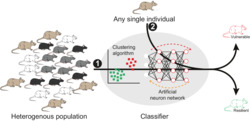

## 1 INTRODUCTION

### 1.1 Background

The past decades have been the stage of a profound change in the conceptualisation of the delineation of so‐called abnormal from adaptive psychology, with a progressive transition from a categorical to a multidimensional approach to the diagnosis of psychiatric disorders (Brooks et al., 2017; Ford et al., 2014; Insel, 2014; Woody & Gibb, 2015), increasingly reliant on transdiagnostic endophenotypes of vulnerability. This ongoing transition at the clinical level has occurred in conjunction with an increasing interest in the preclinical field of substance use disorder (SUD) in the neurobehavioural basis of the individual vulnerability to switch from controlled to persistent, or compulsive, drug seeking and/or taking (Augier et al., 2018; Cannella et al., 2020; Harada et al., 2021; Kasanetz et al., 2010, 2013; Luscher et al., 2020; Pascoli et al., 2018; Pohorala et al., 2021; Radwanska & Kaczmarek, 2012). These developments in both fields warrant the development of new dimension‐based diagnostic or stratification tools aiming objectively and systematically to identify vulnerable and resilient individuals, a necessary step towards the standardisation of translational research in SUD in particular and in psychiatry in general.

Like for many psychiatric disorders, the presence of a triggering factor, such as exposure to a drug in the case of SUD, is not sufficient for the development of the several behaviours, which by the extreme nature of their manifestation alongside the continuum of their respective dimensions, are characteristic of the pathology. This individual vulnerability to transition from controlled, recreational drug use to the compulsive drug seeking and taking behavior that characterizes SUD (APA, 2013) has long been suggested to stem from the interaction between environmental, psychological, neurobiological and behavioural factors (Anthony et al., 1994; Conway et al., 2002; Ersche et al., 2012, 2020; Grant et al., 2001, 2004; Swendsen et al., 2009, 2010). However, it is difficult to identify and study the biobehavioural basis of the factors conferring this vulnerability in humans, not least because such endeavours require the study of large populations across their lifetime in controlled conditions, with little if any opportunity to carry out the invasive manipulations that are necessary to identify the underlying neural and cellular mechanisms.

Over the past two decades, preclinical models have progressively evolved to incorporate the importance of these individual differences, thereby offering unique opportunities to overcome these limitations by using prospective longitudinal studies to investigate the psychological and neural basis of the vulnerability to developing addiction‐like behaviour (Belin‐Rauscent et al., 2016). Indeed, as in humans, all individual rats that regularly self‐administer or seek addictive drugs do not necessarily lose control over drug intake and develop persistent, compulsive drug‐seeking and taking behaviours. In this context, in the early 2000's, a multidimensional model of addiction was developed (Deroche‐Gamonet et al., 2004) based on the intersectionality of specific behavioural characteristics that are the operationalization of DSM‐IV criteria (APA, 1994), namely, increased motivation to take the drug, inability to refrain from drug‐seeking and continued drug use despite knowledge of aversive consequences. This approach enables the identification of divergent trajectories of the transition from controlled to compulsive drug intake in that only 20% of a given population of outbred rats exposed to cocaine eventually displays the three‐behavioural criteria following a prolonged (>60 daily sessions) history of self‐administration. Importantly, rats identified as displaying the 3‐criteria for addictive behaviours also show an increased tendency to escalate their drug intake when access is illimited, and they are prone to relapse following abstinence (Belin et al., 2009), thereby displaying additional behavioural manifestations reminiscent of diagnostic criteria for which they were not selected. The construct and predictive validity of the 3‐criteria model were further substantiated as these differences between vulnerable and resilient rats are not due to a differential cocaine exposure since all rats self‐administer the same amount of drug before being identified as 3 vs 0 criteria (Deroche‐Gamonet et al., 2004). However, while they do not take more cocaine, 3crit rats develop a binge‐like pattern of intake that precedes the transition to addiction (Belin et al., 2009).

The 3‐criteria model has led to several breakthroughs in our understanding of the vulnerability to addiction which together represent a unique success story of translational research. This model first helped establish that impulsivity (Belin et al., 2008) and boredom susceptibility (Belin et al., 2011) confer a vulnerability to switch from controlled to compulsive cocaine intake. In contrast, both sensation seeking, assessed as a greater locomotor response to novelty, and sign tracking, which predict an increased tendency to initiate drug self‐administration and to respond to drug‐paired cues, respectively, were revealed to confer resilience to addiction‐like behaviours (Belin et al., 2008; Fouyssac et al., 2021). These observations in rats have paved the way for studies in humans confirming that the factors associated with recreational cocaine use are dissociable from those specifically associated with the transition to SUD (Ersche et al., 2010). The evidence of a causal relationship between a high impulsivity trait and the subsequent vulnerability to developing compulsive behaviours (Ansquer et al., 2014; Belin et al., 2008) has far‐reaching implications for our understanding of the neural basis of addiction (Besson et al., 2013; Dalley et al., 2007; Fouyssac et al., 2021), which the model helped reveal to be very different to the biological responses to drug exposure. Thus, the tendency to persist in drug taking despite adverse consequences is associated with rigidity, not an exacerbation, as it is the case following single or repeated administrations of drugs, of drug‐induced synaptic plasticity (Kasanetz et al., 2010; Pascoli et al., 2014; Ungless et al., 2001).

This multidimensional approach has since been applied to the study of the neural and psychological basis of the vulnerability to developing alcohol use disorder (AUD) (Jadhav et al., 2017, 2018) or compulsive‐like food seeking (de Jong et al., 2013), illustrating the high translational value of novel preclinical models that encapsulate the multidimensional nature of SUD and the importance of focusing on the individual.

However, these procedures are all dependent on defining a threshold above which a behaviour is deemed maladaptive; strikingly where the cursor should be placed on a continuum to consider a behavior abnormal is a very challenging question, especially at a time of a transition from categorical to dimensional approaches. For example, in the 3‐criteria model, the threshold used for an individual to be deemed positive for each addiction‐like criterion is determined by the unique physical properties of the distribution of the population for one of the three criteria, namely resistance to punishment (measured as infusions during punishment as a percentage of the baseline number of infusions) (Belin et al., 2008, 2011; Deroche‐Gamonet et al., 2004; Piazza & Deroche‐Gamonet, 2013). Along the dimension of compulsiveness, while the majority of any population (60 to 70%) belongs to a log normal distribution ranging from 0–30% resistance, the remaining 30–40% belongs to an abutting normal distribution centred on 85–100% resistance. The bimodal distribution of this dimension, which ranges from noncompulsive to absolutely persistent, compulsive drug self‐administration, offers an objective threshold selection for the associated criterion, but its application to the two other criteria, inability to relinquish drug seeking even in the absence of the drug and high motivation for the drug, which both follow a log‐normal distribution (see Belin & Deroche‐Gamonet, 2012 for review), relies on the assumption that a similar rupture in the continuum exists in them too, which is an inherent limitation. In addition, a distribution‐based threshold selection to ascribe diagnostic scores puts too large an emphasis on the population to which each individual belongs, the physical properties of which eventually contribute almost as much as the individual characteristics themselves to its characterization as ‘addicted‐like’ or ‘resilient’. This thereby precludes the determination of the vulnerability status of a given individual considered independently of a particular cohort, as is the case in humans, a limitation of the underlying approach, which together with the associated need to train large cohorts at once for long periods of time, may have hindered the development of preclinical and/or translational research programmes using this or similar multi‐dimensional preclinical models of addiction.

Recent developments in machine learning may offer unprecedented means to overcome these limitations as they have been suggested to be ideal tools for the refinement of the classification of individuals along dimensions, including psychiatric patients, within subgroups with shared underlying endophenotypes, an approach necessary for the implementation of more effective, personalised therapeutic strategies (Bzdok & Meyer‐Lindenberg, 2018). These approaches also have an advantage over classical statistics (e.g., null hypothesis testing, ANOVAs), because they uncover substructures/subgroups in data without necessarily receiving specific instructions, e.g., in the absence of any a priori hypothesis with regards to the data structure, and yet they endow the classifiers they underlie with the ability systematically to extrapolate patterns learnt from the data with which they are trained to entirely new data sets with individual precision.

Hence, here we used the 3‐criteria multidimensional models for cocaine or alcohol addiction to test the potential of machine‐learning assisted classifiers to identify individuals with or without addiction‐like behaviour in drawing on diagnostic‐relevant dimensions of addiction (APA, 1994, 2013). For this, we subjected the individual scores in each of the three addiction‐like behaviours to different clustering algorithms and then validated the labels using supervised prediction algorithms.

## 2 MATERIALS AND METHODS

### 2.1 Data

Data from four published papers (Belin et al., 2008, 2009, 2011; Fouyssac et al., 2021) were used to assess addiction‐like behavior in cocaine. The data from the first three studies were pooled as a heterogenous cohort of 88 individuals **(All_data_cocaine.csv)** to train the classifier, while the data (*n* = 36, **Cocaine_independent_dataset.csv**) of the most recent publication (Fouyssac et al., 2021) were used as a completely independent dataset to test its generalisability and accuracy. Of the 11 studies published so far using this model (Belin et al., 2008, 2009, 2011; Cannella et al., 2013, 2018, 2020; Deroche‐Gamonet et al., 2004; Fouyssac et al., 2021; Kasanetz et al., 2010, 2013; Pohorala et al., 2021), those selected for the present study were the only ones using footshock as punishment that also provided clear delineation of each of the four criteria groups and easy access to distributions. They also ensured the robustness and generalisability of the classifier that was initially intended as they encompass a large experimental and individual heterogeneity including Sprague Dawley or Lister Hooded rats that nose‐poked or lever pressed for cocaine and were housed in different conditions.

For addiction‐like behaviour for alcohol, data were pooled from two published (Jadhav et al., 2017, 2018) and one unpublished experiment with a cohort of 150 rats **(All_data_alcohol.csv)**.

All analyses were processed using Python 3.8 using Numpy, Pandas and Scikit‐learn packages as well as TensorFlow‐Keras for deep learning methods (Chollet & others, 2015; Pedregosa et al., 2011).

The instrumental responses (i.e., active lever presses/nose pokes) performed in each of the three behavioral tests (termed ‘*raw data’*) were used as the three dimensions injected in the algorithms) namely, increased motivation to take the drug, as measured under a progressive ratio schedule of reinforcement, inability to refrain from drug seeking, as measured during two periods within each daily session during which a discriminative stimulus signals that instrumental responding does not give access to the drug, and maintained drug use despite aversive consequences (compulsivity), measured as the persistence of responding despite punishment of the instrumental response. These dimensions have been shown to represent marginally overlapping, complementary aspects of addiction‐like behaviour (Belin et al., 2008; Deroche‐Gamonet et al., 2004; Jadhav et al., 2017).

**FIGURE 1:**
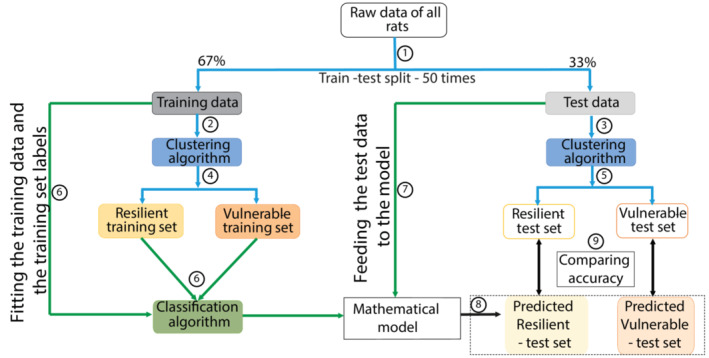
Workflow of the machine learning classifier. The steps are illustrated as numbers in the circles. Clustering algorithms used are Gaussian mixture method and K‐mean/K‐median clustering. Classification algorithms used are K nearest neighbor, logistic regression, support vector machines and artificial neural networks. The blue arrows indicate the clustering algorithms and the green arrows indicate the classification algorithms

The large datasets were split 50 times into 50 different training (67%) and test sets (33%) to avoid a cohort‐driven bias in the clustering of individual rats (Figure 1, ①).

### 2.2 Algorithm

Individuals consuming drugs can be categorized as resilient or vulnerable to the development of SUD, the latter further being distributed along a clinical continuum of severity (Aguilar et al., 2020; Ersche et al., 2020; Morrow & Flagel, 2016), suggesting that any population could be segregated into two clusters. Nevertheless, the optimal cluster number to be used in the classifier was determined by subjecting the 50 training and 50 test sets (i.e., 100 sets) **(Cocaine_cluster_number_files.zip, Alcohol_cluster_number_files.zip)** to the Silhouette algorithm to inform the expected cluster number based on the actual experimental dataset **(cluster_numbers_cocaine.py, cluster_numbers_alcohol.py)** and the one most commonly informed by the silhouette algorithm across 100 iterations, was included as an input in the clustering algorithms of the classifiers tested in the study.

**FIGURE 2:**
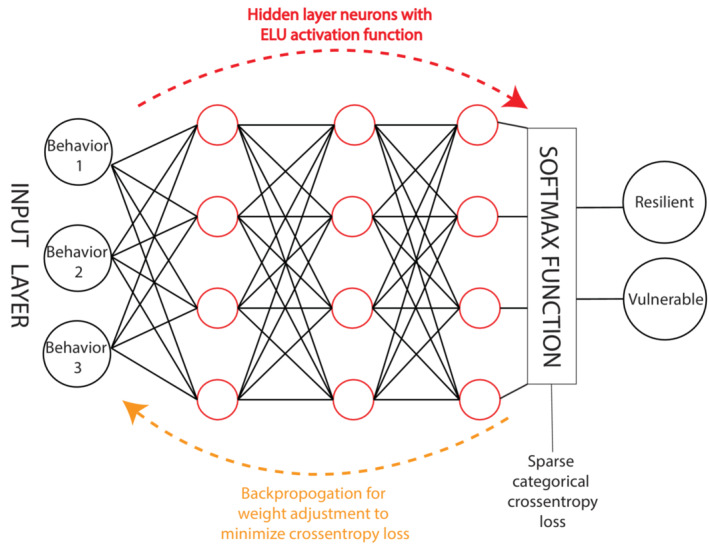
Illustration of an artificial neural network. The hidden layer consists of neurons with the ELU (exponential linear unit) activation function. Feed forward network entails multiple forward passes through the hidden layers. One forward pass consists of consecutive matrix multiplications at each layer by utilizing random weights to initialize the training, which are then adjusted during backpropagation to minimize the cross entropy loss function. Maintained drug use despite aversive consequences, increased motivation to take the drug and inability to refrain from drug seeking during signalled unavailability are represented as behaviour 1, behaviour 2 and behaviour 3, respectively.

**FIGURE 3:**
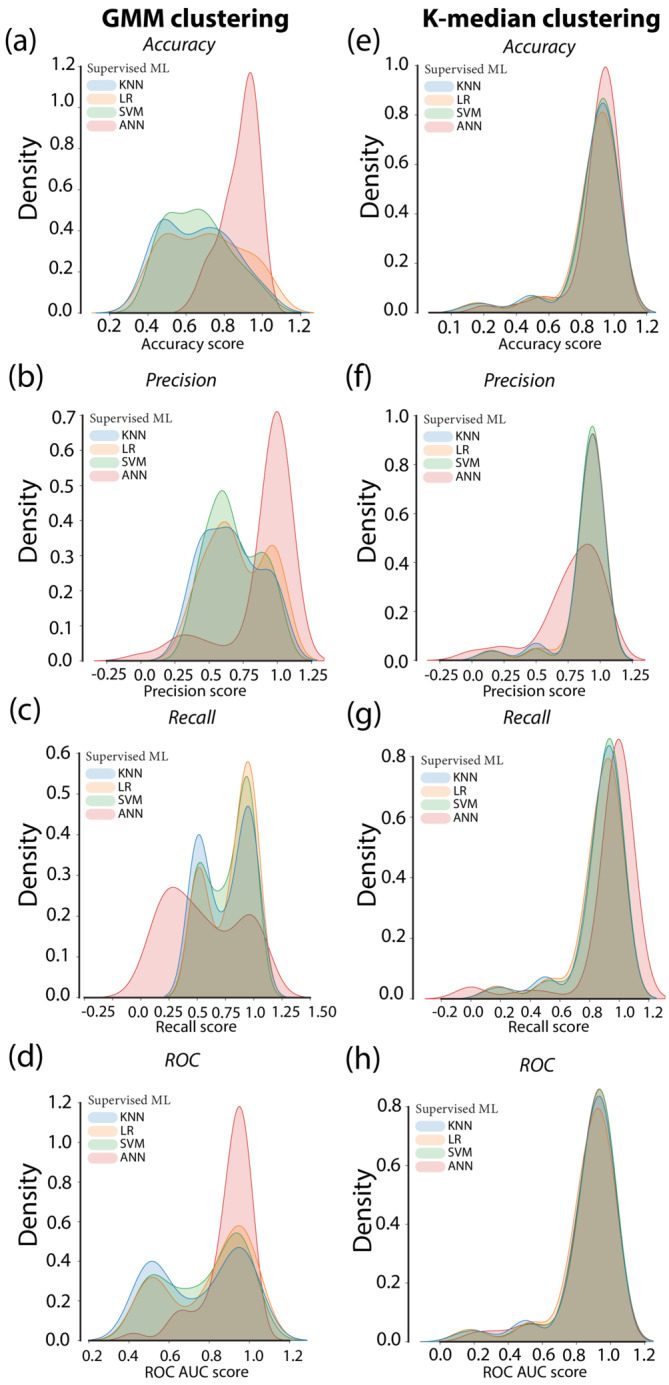
Performance evaluation metrics of the machine learning classifier of the addiction‐like behavior for cocaine in rats. FIGURE 3A–3D depict the accuracy, precision, recall and ROC AUC score, respectively of the GMM clustering‐based classifier followed by four supervised machine learning algorithms. 3E–3H depict the accuracy, precision, recall and ROC AUC score respectively of the K‐median clustering‐based classifier followed by the four supervised machine learning algorithms. GMM: Gaussian mixture method, ML: Machine learning, KNN: K‐nearest neighbor, LR: Logistic regression, SVM: Support vector machines, ANN: Artificial neural networks.

**FIGURE 4:**
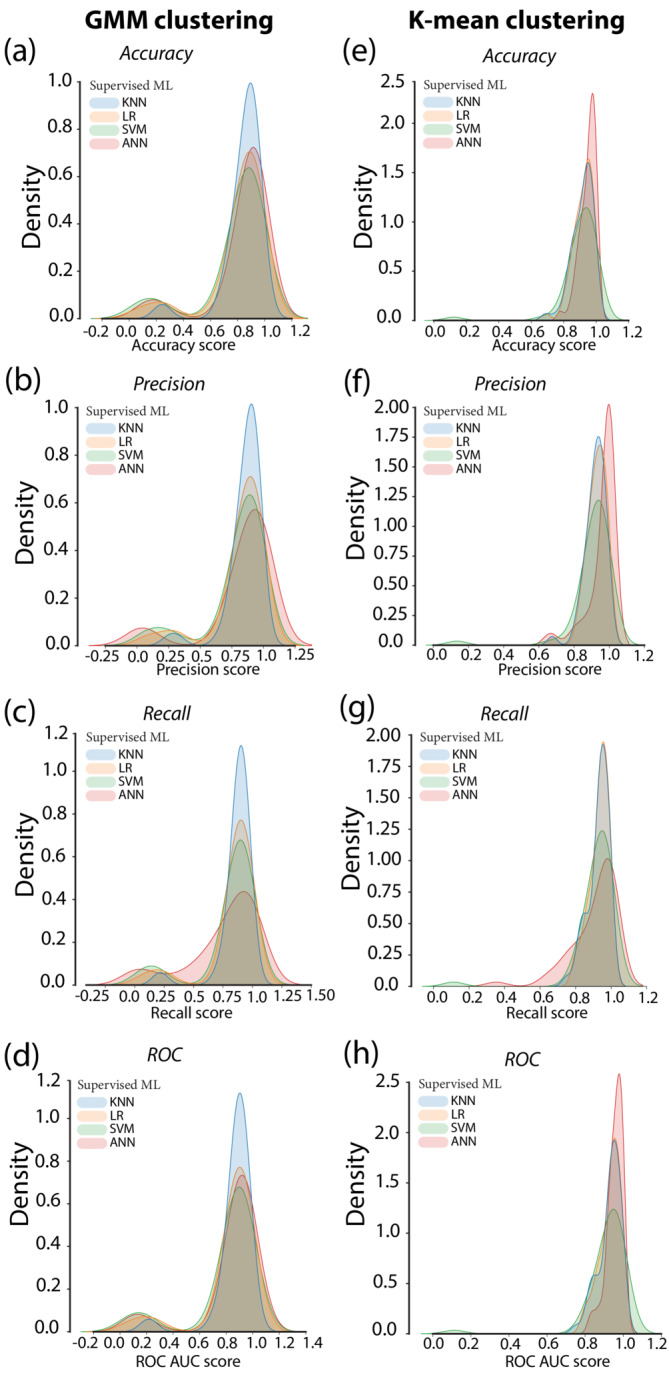
Performance evaluation metrics of the machine learning classifier of the addiction‐like behavior for alcohol in rats. FIGURE 4A‐D depict the accuracy, precision, recall and ROC AUC scores respectively of the GMM clustering based classifier followed by four supervised machine learning algorithms. 4E–4H depict the accuracy, precision, recall and ROC AUC scores respectively of the K‐mean clustering‐based classifier followed by four supervised machine learning algorithms. GMM: Gaussian mixture method, ML: Machine learning, KNN: K nearest neighbor, LR: Logistic regression, SVM: Support vector machines, ANN: Artificial neural networks.

**FIGURE 5:**
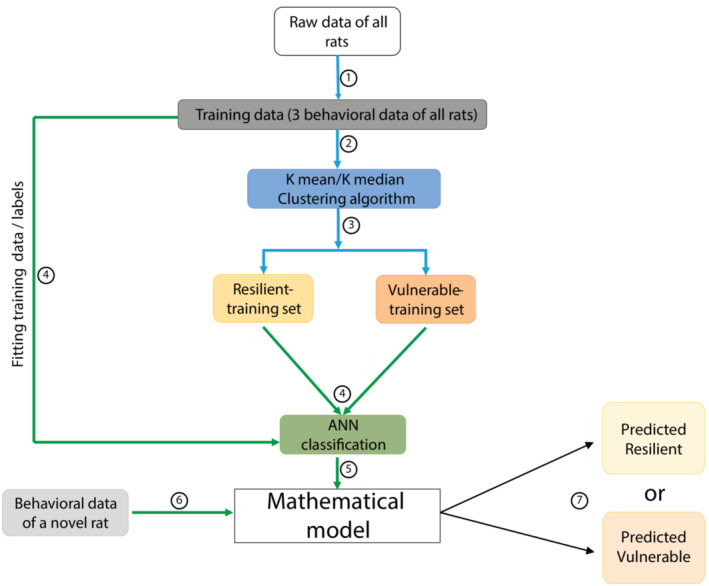
Flowchart for the labelling of any future rat as resilient or vulnerable to develop substance use disorder. The steps are illustrated as numbers in the circles. Having established that the classifier based on K‐median/K‐mean clustering followed by ANN gives the best predictive accuracy, the addiction vulnerability status of a single rat irrespective of the cohort it is trained with. The blue arrows indicate the clustering algorithms and the green arrows indicate the classification algorithms. ANN: Artificial neural network

Behavioural data of a single pair of Training and Test set was subjected to unsupervised clustering algorithms (Figure 1, ②,③) (namely Gaussian mixture model (GMM) (Reynolds, 2009) or K‐mean/K‐median clustering (Forgy, 1965)) (SOM) to determine resilient and vulnerable rats in both sets (Figure 1, ④,⑤).

We used four supervised classification algorithms (SOM), namely K‐nearest neighbour (KNN) (Forgy, 1965), logistic regression (LR) (Cramer, 2002), support vector machines (SVM) (Pedregosa et al., 2011) and artificial neural networks (ANN) (Zou et al., 2008) (Figure 2) to fit the behavioural data of the Training Set and the labels assigned by the clustering algorithm to generate a mathematical model that best explains the behavioural data and the labels of the rats in the Training Set (Figure 1, ⑥). For ANN, increasing numbers of hidden layers were used (5, 50 and 500), keeping the number of neurons in each layer constant, to test both a potential tendency to overfit and the ability of the algorithm to accommodate larger sample sizes in the future. Then, to predict the labels of the rats belonging to the Test set, their behavioural data were submitted to the mathematical model (Figure 1, ⑦) generated by each of the four supervised classification algorithms.

When submitted to these mathematical models, the behavioural data of the Test Set is used to ascribe a resilient or vulnerable label to each rat of the Test Set (Figure 1, ⑧) (Pedregosa et al., 2011). Thus, each rat in the Test set is ascribed two labels, one by the unsupervised‐clustering algorithm and one by a particular supervised‐prediction algorithm. The goal of this approach is to determine the unsupervised clustering–supervised prediction combination that yields overlapping labels for the Test Set rats (Figure 1, ⑨).

The labels assigned to the Test set rats by the clustering algorithm (considered here as true labels) and the predicted labels of the same rats by a supervised‐prediction algorithm can be represented in a classification matrix (Table 1).

**TABLE 1:** Classification matrix

	Vulnerable (supervised classification‐based predictions‐ test dataset)	Resilient (supervised classification‐based predictions ‐ test dataset)
Vulnerable (unsupervised clustering: Test dataset)	True vulnerable (TV)	False resilient (FR)
Resilient (unsupervised clustering: Test dataset)	False vulnerable (FV)	True resilient (TR)

Accuracy ([TV + TR]/[TV + TR + FV + FR]), precision (TV/[TV + FV]), recall (TV/[TV + FR]) and ROC AUC (Kumar & Indrayan, 2011) scores were calculated from the classification matrix (SOM).

Each pair of Training and Test Set were subjected to this pipeline four times, i.e., GMM‐clustering followed by the four supervised prediction algorithms. As mentioned previously, there were 50 pairs of Training and Test sets, so that for each combination of GMM clustering–supervised prediction algorithm, 50 iterations were processed, resulting in 50 accuracy, precision, recall and AUC ROC scores. Similarly, the same procedure was followed for K‐median/K‐mean clustering followed by four supervised prediction algorithms. Results are depicted as kernel density estimates of the probability density function of these 50 iterations for all four performance evaluation metrics for each combination of unsupervised clustering–supervised prediction algorithm.

## 3 RESULTS

For addiction‐like behaviour for cocaine, the Silhouette score revealed the optimal number of clusters was ‘2’ in 88% (Training sets–K‐median clustering), 76% (Test sets–K‐median clustering), 74% (Training sets‐GMM clustering) and 76% (Test sets–GMM clustering), while the second most commonly suggested cluster number ranged from 3 to 6. Similarly, for addiction‐like behaviour for alcohol, the optimal number of clusters was ‘2’ in 74% (Training sets–K‐mean clustering), 78% (Test sets–K‐mean clustering), 96% (Training sets‐GMM clustering) and 70% (Test sets–GMM clustering), while the second most commonly suggested cluster number ranged from 3 to 6. This analysis confirmed that two clusters should be used in subsequent analyses.

For addiction‐like behaviour for cocaine, K‐median‐KNN, K‐median‐LR and K‐median‐SVM (**Kmedian_cocaine.py**) classifiers yielded similar scores (Table 2A, Figure 3) that were overall superior to GMM‐KNN, GMM‐LR and GMM‐SVM (**GMM_cocaine.py**) classifiers with regard to median accuracy, precision, recall and ROC‐AUC scores as well as the proportion of iterations reaching the top ten percentile, respectively (Table 2B, Figure 4). The K‐median‐ANN classifier and GMM–ANN classifier gave similar median accuracy and ROC–AUC scores and resulted in a similar proportion of these scores being in the top ten percentile (Tables 2A, 2B, Figure 3).

**TABLE 2A:** Classifier based on K‐median clustering followed by supervised algorithm‐based predictions for addiction‐like behavior for cocaine

	Accuracy		Precision		Recall		ROC AUC score	
	Median	Scores in top 10 percentile	Median	Scores in top 10 percentile	Median	Scores in top 10 percentile	Median	Scores in top 10 percentile
KNN	0.93	58%	0.93	72%	0.92	58%	0.92	58%
LR	0.92	56%	0.93	72%	0.91	52%	0.91	52%
SVM	0.93	60%	0.92	76%	0.93	60%	0.93	60%
ANN^5^	0.86	26%	0.7	18%	1	72%	0.83	26%
ANN^50^	0.93	52%	0.80	36%	1	88%	0.9	48%
ANN^500^	0.96	48%	0.9	42%	1	92%	0.95	68%

**TABLE 2B:** Classifier based on GMM clustering followed by supervised algorithm‐based predictions for addiction‐like behavior for cocaine

	Accuracy		Precision		Recall		ROC AUC score	
	Median	Scores in top 10 percentile	Median	Scores in top 10 percentile	Median	Scores in top 10 percentile	Median	Scores in top 10 percentile
KNN	0.67	16%	0.75	28%	0.88	48%	0.88	48%
LR	0.73	6%	0.67	22%	0.87	46%	0.87	46%
SVM	0.68	10%	0.66	22%	0.89	48%	0.89	48%
ANN^5^	0.9	34%	1	52%	0.36	22%	0.84	42%
ANN^50^	0.93	60%	1	74%	0.67	38%	0.94	62%
ANN^500^	0.93	56%	1	80%	0.6	24%	0.96	74%

GMM: Gaussian mixture model, SML: Supervised Machine Learning, KNN: K nearest neighbor, LR: Logistic Regression, SVM: Support Vector Machines, ANN: Artificial Neural Networks. ANN^5^, ANN^50^, ANN^500^: numbers indicate the number of hidden layers of the ANN.

**TABLE 3A:** Classifier based on K‐mean clustering followed by supervised algorithm‐based predictions for addiction‐like behavior for alcohol

	Accuracy		Precision		Recall		ROC AUC score	
	Median	Scores in top 10 percentile	Median	Scores in top 10 percentile	Median	Scores in top 10 percentile	Median	Scores in top 10 percentile
KNN	0.92	66%	0.93	68%	0.94	78%	0.94	78%
LR	0.93	62%	0.93	68%	0.94	72%	0.94	72%
SVM	0.93	62%	0.93	68%	0.93	76%	0.93	76%
ANN^5^	0.92	60%	0.93	60%	0.94	60%	0.93	66%
ANN^50^	0.96	76%	1	74%	0.94	60%	0.96	88%
ANN^500^	0.96	82%	1	86%	0.97	64%	0.97	90%

For addiction‐like behaviour for alcohol, K‐mean‐KNN, K‐mean‐LR, K‐mean‐SVM and K‐mean‐ANN (**Kmean_alcohol.py**) classifiers yielded similar scores for all the performance evaluation metrics (Table 3A, Figure 4) that were overall superior to those obtained by GMM‐KNN, GMM‐LR, GMM‐SVM and GMM‐ANN (**GMM_alcohol.py**) classifiers (Table 3B, Figure 4).

**TABLE 3B:** Classifier based on GMM clustering followed by supervised algorithm‐based predictions for addiction‐like behavior for alcohol

	Accuracy		Precision		Recall		ROC AUC score	
	Median	Scores in top 10 percentile	Median	Scores in top 10 percentile	Median	Scores in top 10 percentile	Median	Scores in top 10 percentile
KNN	0.87	40%	0.90	50%	0.89	48%	0.89	48%
LR	0.87	42%	0.89	46%	0.89	46%	0.89	46%
SVM	0.86	40%	0.88	46%	0.89	44%	0.89	44%
ANN^5^	0.87	40%	0.88	46%	0.85	32%	0.89	46%
ANN^50^	0.88	36%	0.91	56%	0.82	38%	0.89	44%
ANN^500^	0.92	54%	0.91	64%	0.9	50%	0.92	60%

GMM: Gaussian mixture model, SML: Supervised Machine Learning, KNN: K nearest neighbor, LR: Logistic Regression, SVM: Support Vector Machines, ANN: Artificial Neural Networks. ANN^5^, ANN^50^, ANN^500^: numbers indicate the number of hidden layers of the ANN.

The performance of the K‐median‐ and GMM‐ANN or K‐mean‐ and GMM‐ANN classifiers was further improved by an increase in the number of hidden layer neurons used in the ANN (Tables 2B and 3B). The ensuing increase in accuracy thereby demonstrates the ability of the K‐median/K‐mean‐ANN classifiers to accommodate larger sample sizes and/or more dimensions, a feature that is not reflective of overfitting since back propagation and early stopping processes were included in the ANN (Caruana et al., 2001).

Together, these results demonstrate that a classifier based on K‐median/K‐mean followed by ANN is the most robust and future‐ and dimension expansion‐proof approach to accurately predict whether a single rat is vulnerable or resilient as assessed in our multisymptomatic model with great heuristic value with regards to the clinical definition of SUD (Figure 5).

To cross validate the classifier, the entire datasets related to cocaine and alcohol addiction‐like behaviour (*n* = 88, *n* = 150, respectively) were subjected to the K‐median (**All_cocaine_Kmedian.py**)/K‐mean clustering (**All_alcohol_Kmean.py**). All the rats originally characterized as 0 or 3crit in their respective cohorts were correctly labelled as resilient or vulnerable, respectively, revealing an absolute intersection (Tables 4A and 4B) (**Cocaine_crit_correspondence.xlsx, Alcohol_crit_correspondence.xlsx**).

**TABLE 4A:** Addiction‐like behavior for cocaine

	0Crit in the original cohort	3Crit in the original cohort
Resilient (K median clustering)	36	0
Vulnerable (K median clustering)	0	15

**TABLE 4B:** Addiction‐like behavior for alcohol

	0Crit in the original cohort	3Crit in the original cohort
Resilient (K mean clustering)	64	0
Vulnerable (K mean clustering)	0	18

Finally, in order to establish the predictive potential of the classifiers we developed, we applied them to a completely new dataset (Fouyssac et al., 2021) that consists of the 3‐criteria behavioural scores of a cohort of 36 rats housed either in a standard (two individuals in a standard cage) or an enriched environment. While replicating previous findings that environmental enrichment decreases the tendency to self‐administer cocaine (Bardo et al., 2001; Puhl et al., 2012), this study demonstrated that rats housed in an enriched environment were more vulnerable to developing addiction‐like behaviour than rats raised in a standard environment (Fouyssac et al., 2021) in that all the 3crit rats identified in this heterogeneous cohort came from the former. In line with the original study, none of the rats from the standard housing group were labelled as vulnerable by the classifiers, while those identified as vulnerable overlapped with 100% accuracy with those identified as 3crit that came from the enriched environment (**EEES.xls**) (Fouyssac et al., 2021).

## 4 DISCUSSION

The next frontier in addiction research lies in understanding the environmental, psychological and biological mechanisms that mediate, in vulnerable individuals, the transition from controlled drug intake to the compulsive seeking and taking characteristic of SUD. Behavioural procedures that enable the study, under controlled conditions, of individual trajectories from a drug naïve state to the development of addiction‐like behaviour over the course of drug self‐administration have only started to demonstrate their utility in our understanding of the mechanisms of individual vulnerability to addiction (Belin et al., 2008, 2009; Besson et al., 2013; Deroche‐Gamonet et al., 2004; Fouyssac et al., 2021; Jadhav et al., 2017, 2018). These procedures have hitherto been limited by a lack of an objective diagnosis strategy, i.e., one that is not influenced by the physical data‐distribution properties of the cohort to which an individual belongs, thereby resulting in the unwarranted need to train large cohorts of animals at any given time and detracting the approach from the individual‐centred diagnosis in humans.

In this study, we drew on large datasets produced over the past two decades, to develop new machine‐learning asssisted classifiers for cocaine or alcohol addiction‐like behaviour that characterize with high accuracy single individuals, irrespective of the cohort to which they belong, as resilient or vulnerable.

The role of clustering algorithms is to identify data points in a multidimensional space that are closer to one another than they are to any other data point in the cloud (Fung, 2001). In many real‐life situations, the labels of such data‐points are obvious, e.g., males vs females for biological differences, or voted for/against Brexit. In these situations, data clustering is not necessary. However, ascribing labels, such as those to determine if an individual meets the criteria of addiction‐like behaviour, cannot be informed by natural dichotomic population segregation. This requires structuring a multidimensional space into delineated subspaces which can be used to ascribe a specific label to each individual constituent of the cluster and to train supervised classification algorithms in order to successfully predict the label, i.e., the specific cluster to which they most likely belong of a single individual whose data has never been used to train the classification algorithm.

The first step of such an algorithm's development was to objectively determine the cluster number to structure the multidimensional cloud to accommodate the physical properties of the data and the objective of the classifier. In real life, individuals can be categorized as vulnerable or resilient, thereby suggesting that any experimental population could be segregated into two clusters (Aguilar et al., 2020; Ersche et al., 2020; Morrow & Flagel, 2016). Nevertheless, a data‐driven approach was used to ensure that such a dichotomy was present in the experimental datasets. The Silhouette algorithm ran on all the datasets used here systematically revealed that the multidimensional space of the datasets was predominantly structured around two clusters, an outcome that is compatible with the prerequisite of the algorithm: to segregate two subpopulations from heterogeneous groups, namely vulnerable and resilient individuals. This also provided an unbiased threshold for the various cluster analyses (GMM and K‐median/K‐mean) used in the several potential classifiers tested in this study.

Identification of resilient or vulnerable rats in the 3‐criteria model was hitherto based on the bimodal distribution of each population for resistance to punishment (Belin et al., 2008; Deroche‐Gamonet et al., 2004; Jadhav et al., 2017) which comprises a large log‐normally distributed subpopulation of non‐compulsive rats (60–70% population) tailed by an independent, normally distributed population of compulsive rats (30–40% population). Since GMM‐clustering can fit bi/multimodal data distributions (Lubke & Muthen, 2005), it was originally used to assimilate such physical properties on which depends the selection threshold for addiction‐like behaviour. However, the GMM‐based classifier did not yield outputs superior to the K‐median/K‐mean‐based classifier. This surprising outcome can be due to the fact that a GMM classifier, in contrast with the strategy we developed to apply the 30–40% threshold to the other two criteria, each characterized by a log‐normal distribution, uses differential densities across quartiles in each variable independently to develop the classifier.

K‐median/K‐mean clustering, which is based on the Euclidean distance between the data‐points in a three‐dimensional vector space that plots the number of responses along three axes representing three different psychological constructs, was revealed to be the superior clustering method to accurately and consistently ascribe labels of resilience vs vulnerability. Not only are K‐median/K‐mean algorithms easy to implement, but they are scalable and can be used to separate nonlinearly separable data. These properties were exploited to develop a robust and universal classifier. Thus, the same clustering algorithms were applied to 50 independent sets drawn from a large dataset comprising data from experiments carried out in different laboratories, on different strains (Sprague–Dawley (Belin et al., 2009, 2011) or Lister‐Hooded (Belin et al., 2008) for cocaine addiction‐like behaviour or Wistar rats (Jadhav et al., 2017, 2018)) for alcohol addiction‐like behaviour that differed in addiction relevant traits (McDermott & Kelly, 2008) and using different instrumental responses (nose‐pokes (Belin et al., 2009, 2011) or lever presses (Belin et al., 2008)). The ability of the classifier to survive randomization tests and to generalize across response modalities and strains demonstrates its potential use across a large repertoire of experimental idiosyncrasies that may reflect the behavioural heterogeneities observed by clinicians when a diagnosis is warranted. Furthermore, the ability of the K‐median/K‐mean‐supervised algorithm classifiers to accurately identify rats as being vulnerable to addiction or resilient from a completely different dataset generated with a heterogeneous cohort exposed to very different housing conditions to those used in the experiments exploited for the training datasets (Fouyssac et al., 2021) indicates that these tools can be deployed across many diverse experimental conditions.

Nevertheless, the same classifier could not be generalized from addiction‐like behavior for one drug to another drug. While the K‐median clustering‐based algorithm used for addiction‐like behaviour for cocaine systematically yielded the right vulnerability/resilient labels, even when applied to a dataset never used in its development (the enriched environment experiment (Fouyssac et al., 2021)), it was suboptimal in the case of addiction‐like behaviour for alcohol, the best classifier for which was based on K‐mean clustering. The lack of generalizability of a given classifier across drugs is further evidence of construct and predictive validity since AUD and SUD are independent diagnoses in humans and they have long been shown to involve different psychological and neurobiological mechanisms (Nestler, 2005). In addition, while the 3‐criteria model for cocaine relies on the assessment of compulsive cocaine intake (consummatory conflated with preparatory responses as is the case under fixed‐ratio schedules of reinforcement) (Belin‐Rauscent et al., 2016), the 3‐criteria model for alcohol is based on the assessment of the compulsive nature of a seeking response in a chained schedule where lever pressing results in the procurement of alcohol, the ensuing consumption of which occurs in a dedicated magazine, involving a set of behavioural responses independent of the instrumental component of the chain. Considering how neurally and psychologically dissociable preparatory and consummatory responses are (Blackburn et al., 1989; Everitt, 1990), it was not expected that a single classifier could be used across measures of compulsive taking and seeking. However, it will be interesting to test in future studies if the alcohol‐specific K‐mean classifier can be applied to compulsive cocaine seeking data, as measured under second‐order or seeking‐taking heterogeneous chained schedules of reinforcement, which dissociate seeking from taking/consummatory responses for orally (Giuliano et al., 2021, 2022) and intravenously administered drugs (Everitt et al., 2018; Fouyssac et al., 2022; Murray et al., 2012; Pelloux et al., 2015).

Irrespective of the outcomes of these future studies, a one‐size‐fit‐all approach does seem to be not an optimal expectation, and further research is needed to consider the ability of such a classifier to accommodate the potential differences in the multidimensional relationship between addiction‐related behavioural criteria that may exist between males and females, which, to the best of our knowledge, have not yet been experimentally investigated. Another important avenue for future research is to identify mathematical tools that will enable the introduction of dimensionality within the categories that are now identified accurately with the K‐mean/K‐median classifiers. The 3‐Criteria model was designed to have construct validity with regards to the diagnosis strategy of DSM‐IV (APA, 1994), i.e., prior to the development of the RDoC (Brooks et al., 2017). Nevertheless, the approach we had then developed embedded a dimensional aspect, in that rats were not only stratified as showing 0 criterion or 3 criteria (deemed resilient and showing addiction‐like behaviour, respectively), but 30–40% of any population was also stratified as showing 1 or 2 criteria. In some studies, 1crit and 2crit rats have been considered similar to 0crit and 3crit, respectively (Cannella et al., 2018; Domi et al., 2019; Jadhav et al., 2017), but molecular data, at least for cocaine addiction‐like behaviour, support the notion that 1 and 2 criteria rats represent an intermediate stage that is different to 0 and 3 criteria rats.

As all the resilient rats identified by our classifier included 0crit, most of the 1crit and no 3crit rats, whereas all the rats identified as vulnerable included 3crit and most of the 2crit but no 0crit rats (**Cocaine_crit_correspondence.xlsx, Alcohol_crit_correspondence.xlsx**), it can be suggested that the present classifier does not yet provide the dimensional granularity necessary to distinguish several levels of severity (2crit vs 3crit) within the vulnerable population, thereby warranting further research to determine whether the addition of endophenotypes (Belin et al., 2008, 2011; Jadhav et al., 2017, 2018) to our classifiers will enable them to fully comply with the dimensional nature of the debilitating condition that is SUD. This could contribute to the several initiatives to identify clinically relevant subtypes of SUD (Leggio et al., 2009) through cluster analysis of patients to better capture the clinical heterogeneity (Blanco et al., 2013; Herzig et al., 2015; Kupfer et al., 2008; Kwako et al., 2016, 2019) with the aim of advancing personalized medicine (Mann & Hermann, 2010; Witkiewitz et al., 2019).

### 4.1 Conclusion

The present machine learning‐based classifiers represent a unique tool to objectively identify whether a single experimental subject is resilient or vulnerable to cocaine or alcohol addiction‐like behaviour (Figure 5). The ability conferred by such a tool to consider a single individual irrespective of the experimental cohort to which it belongs (and the associated experimental conditions) bridges a new frontier in the study of the individual vulnerability to developing SUD, bringing the focus back on the individual, as it is the case in humans. It can be boldly envisioned that, with the advent of large data sets in humans from imaging, genomics and proteomic approaches, a successful back‐translation strategy could see the application of such machine learning‐assisted tools to the personalized diagnosis of clinical populations.

## FUNDING INFORMATION

The work of David Belin is supported by a Programme Grant from the Medical Research Council to Barry Everitt, DB, Amy Milton, Jeffrey Dalley and Trevor Robbins (MR/N02530X/1). The work of Veronique Deroche‐Gamonet was supported by ANR‐13‐NEUR‐0002‐01 (ERA‐NET NEURON II), ANR‐10‐LABX‐43 (LABEX BRAIN), ANR‐10‐EQX‐008‐1 (EquipEx OptoPath™), INSERM and Université de Bordeaux. The work of Benjamin Boutrel is supported by a Swiss National Science Foundation grant (310030_185192).

Kshitij S Jadhav is the recipient of the Swiss Government Excellence Fellowship (2015–2018), Doc mobility Fellowship (2018–2019) and Early Post Doc mobility Fellowship (2021–2022) from the Swiss National Science Foundation.

## CONFLICTS OF INTEREST

The authors declare no competing financial interests.

## AUTHOR CONTRIBUTION

The author Kshitij S Jadhav developed the algorithms and wrote the codes. Boury‐Jamot Benjamin provided substantial technical inputs for the code. Kshitij S Jadhav, David Belin, Veronique Deroche‐Gamonet and Benjamin Boutrel wrote the manuscript.

### PEER REVIEW

The peer review history for this article is available at https://publons.com/publon/10.1111/ejn.15839.

## Supporting information


**Data S1.** Supporting Information (SOM)

Supporting Information

Supporting Information

Supporting Information

Supporting Information

Supporting Information

Supporting Information

Supporting Information

Supporting Information

Supporting Information

Supporting Information

Supporting Information

Supporting Information

Supporting Information

Supporting Information

Supporting Information

Supporting Information

## Data Availability

The python code for all the algorithms and the raw data to test the results of this analysis are uploaded as Supporting Information for this manuscript.

## References

[ejn15839-bib-0001] Aguilar, M. A. , Cannella, N. , Ferragud, A. , & Spanagel, R. (2020). Editorial: Neurobehavioural mechanisms of resilience and vulnerability in addictive disorders. Frontiers in Behavioral Neuroscience, 14, 644495. 10.3389/fnbeh.2020.644495 33551770 PMC7855698

[ejn15839-bib-0002] Ansquer, S. , Belin‐Rauscent, A. , Dugast, E. , Duran, T. , Benatru, I. , Mar, A. C. , Houeto, J. L. , & Belin, D. (2014). Atomoxetine decreases vulnerability to develop compulsivity in high impulsive rats. Biological Psychiatry, 75, 825–832. 10.1016/j.biopsych.2013.09.031 24252357

[ejn15839-bib-0003] Anthony, J. C. , Warner, L. A. , & Kessler, R. C. (1994). Comparative epidemiology of dependence on tobacco, alcohol, controlled substances, and inhalants: Basic findings from the National Comorbidity Survey. Experimental and Clinical Psychopharmacology, 2, 244–268. 10.1037/1064-1297.2.3.244

[ejn15839-bib-0004] APA . (1994). Diagnostic and statistical manual of mental disorders ‐ DSM IV. American Psychiatric Association.

[ejn15839-bib-0005] APA . (2013). The diagnostic and statistical manual of mental disorders: DSM 5. bookpointUS. 10.1590/s2317-17822013000200017 24413388

[ejn15839-bib-0006] Augier, E. , Barbier, E. , Dulman, R. S. , Licheri, V. , Augier, G. , Domi, E. , Barchiesi, R. , Farris, S. , Natt, D. , Mayfield, R. D. , Adermark, L. , & Heilig, M. (2018). A molecular mechanism for choosing alcohol over an alternative reward. Science, 360, 1321–1326. 10.1126/science.aao1157 29930131

[ejn15839-bib-0007] Bardo, M. T. , Klebaur, J. E. , Valone, J. M. , & Deaton, C. (2001). Environmental enrichment decreases intravenous self‐administration of amphetamine in female and male rats. Psychopharmacology, 155, 278–284. 10.1007/s002130100720 11432690

[ejn15839-bib-0008] Belin, D. , Balado, E. , Piazza, P. , & Deroche‐Gamonet, V. (2009). Pattern of intake and drug craving predict the development of cocaine addiction‐like behavior in rats. Biological Psychiatry, 65, 863–868. 10.1016/j.biopsych.2008.05.031 18639867

[ejn15839-bib-0009] Belin, D. , Berson, N. , Balado, E. , Piazza, P. V. , & Deroche‐Gamonet, V. (2011). High‐novelty‐preference rats are predisposed to compulsive cocaine self‐administration. Neuropsychopharmacology, 36, 569–579. 10.1038/npp.2010.188 20980989 PMC3055686

[ejn15839-bib-0010] Belin, D. , & Deroche‐Gamonet, V. (2012). Responses to novelty and vulnerability to cocaine addiction: Contribution of a multi‐symptomatic animal model. Cold Spring Harbor Perspectives in Medicine, 2, a011940. 10.1101/cshperspect.a011940 23125204 PMC3543096

[ejn15839-bib-0011] Belin, D. , Mar, A. C. , Dalley, J. W. , Robbins, T. W. , & Everitt, B. J. (2008). High impulsivity predicts the switch to compulsive cocaine‐taking. Science, 320, 1352–1355. 10.1126/science.1158136 18535246 PMC2478705

[ejn15839-bib-0012] Belin‐Rauscent, A. , Fouyssac, M. , Bonci, A. , & Belin, D. (2016). How preclinical models evolved to resemble the diagnostic criteria of drug addiction. Biological Psychiatry, 79, 39–46. 10.1016/j.biopsych.2015.01.004 25747744 PMC4702261

[ejn15839-bib-0013] Besson, M. , Pelloux, Y. , Dilleen, R. , Theobald, D. , Lyon, A. , Belin‐Rauscent, A. , Robbins, T. W. , Dalley, J. W. , Everitt, B. J. , & Belin, D. (2013). Cocaine Modulation of Frontostriatal Expression of Zif268, D2, and 5‐HT2c Receptors in High and Low Impulsive Rats. Neuropsychopharmacology, 38, 1963–1973. 10.1038/npp.2013.95 23632436 PMC3746704

[ejn15839-bib-0014] Blackburn, J. R. , Phillips, A. G. , Jakubovic, A. , & Fibiger, H. C. (1989). Dopamine and preparatory behavior: II. A Neurochemical Analysis. Behavioral Neuroscience, 103, 15–23. 10.1037/0735-7044.103.1.15 2923667

[ejn15839-bib-0015] Blanco, C. , Krueger, R. F. , Hasin, D. S. , Liu, S. M. , Wang, S. , Kerridge, B. T. , Saha, T. , & Olfson, M. (2013). Mapping common psychiatric disorders: Structure and predictive validity in the national epidemiologic survey on alcohol and related conditions. JAMA Psychiatry, 70, 199–208. 10.1001/jamapsychiatry.2013.281 23266570 PMC3777636

[ejn15839-bib-0016] Brooks, S. J. , Lochner, C. , Shoptaw, S. , & Stein, D. J. (2017). Using the research domain criteria (RDoC) to conceptualize impulsivity and compulsivity in relation to addiction. Progress in Brain Research, 235, 177–218. 10.1016/bs.pbr.2017.08.002 29054288

[ejn15839-bib-0017] Bzdok, D. , & Meyer‐Lindenberg, A. (2018). Machine learning for precision psychiatry: Opportunities and challenges. Biological Psychiatry: Cognitive Neuroscience and Neuroimaging, 3, 223–230. 10.1016/j.bpsc.2017.11.007 29486863

[ejn15839-bib-0018] Cannella, N. , Cosa‐Linan, A. , Buchler, E. , Falfan‐Melgoza, C. , Weber‐Fahr, W. , & Spanagel, R. (2018). In vivo structural imaging in rats reveals neuroanatomical correlates of behavioral sub‐dimensions of cocaine addiction. Addiction Biology, 23, 182–195. 10.1111/adb.12500 28231635

[ejn15839-bib-0019] Cannella, N. , Cosa‐Linan, A. , Takahashi, T. , Weber‐Fahr, W. , & Spanagel, R. (2020). Cocaine addicted rats show reduced neural activity as revealed by manganese‐enhanced MRI. Scientific Reports, 10, 19353. 10.1038/s41598-020-76182-3 33168866 PMC7653042

[ejn15839-bib-0020] Cannella, N. , Halbout, B. , Uhrig, S. , Evrard, L. , Corsi, M. , Corti, C. , Deroche‐Gamonet, V. , Hansson, A. C. , & Spanagel, R. (2013). The mGluR2/3 agonist LY379268 induced anti‐reinstatement effects in rats exhibiting addiction‐like behavior. Neuropsychopharmacology, 38, 2048–2056. 10.1038/npp.2013.106 23624743 PMC3746689

[ejn15839-bib-0021] Caruana, R. , Lawrence, S. , & Giles, L. (2001). Overfitting in neural nets: Backpropagation, conjugate gradient, and early stopping. Advances in Neural Information Processing Systems, 13, 402–408.

[ejn15839-bib-0022] Chollet, F. & others . (2015). Keras. GitHub .

[ejn15839-bib-0023] Conway, K. P. , Swendsen, J. D. , Rounsaville, B. J. , & Merikangas, K. R. (2002). Personality, drug of choice, and comorbid psychopathology among substance abusers. Drug and Alcohol Dependence, 65, 225–234. 10.1016/S0376-8716(01)00168-5 11841894

[ejn15839-bib-0024] Cramer, J. S. (2002). The origins of logistic regression. Tinbergen Institute Discussion Papers, 4, 02–119.

[ejn15839-bib-0025] Dalley, J. W. , Fryer, T. D. , Brichard, L. , Robinson, E. S. , Theobald, D. E. , Laane, K. , Pena, Y. , Murphy, E. R. , Shah, Y. , Probst, K. , Abakumova, I. , Aigbirhio, F. I. , Richards, H. K. , Hong, Y. , Baron, J. C. , Everitt, B. J. , & Robbins, T. W. (2007). Nucleus accumbens D2/3 receptors predict trait impulsivity and cocaine reinforcement. Science, 315, 1267–1270. 10.1126/science.1137073 17332411 PMC1892797

[ejn15839-bib-0026] de Jong, J. W. , Meijboom, K. E. , Vanderschuren, L. J. , & Adan, R. A. (2013). Low control over palatable food intake in rats is associated with habitual behavior and relapse vulnerability: Individual differences. PLoS ONE, 8, e74645. 10.1371/journal.pone.0074645 24058616 PMC3769238

[ejn15839-bib-0027] Deroche‐Gamonet, V. , Belin, D. , & Piazza, P. V. (2004). Evidence for addiction‐like behavior in the rat. Science, 305, 1014–1017. 10.1126/science.1099020 15310906

[ejn15839-bib-0028] Domi, A. , Stopponi, S. , Domi, E. , Ciccocioppo, R. , & Cannella, N. (2019). Sub‐dimensions of alcohol use disorder in alcohol preferring and non‐preferring rats, a comparative study. Frontiers in Behavioral Neuroscience, 13, 3. 10.3389/fnbeh.2019.00003 30760988 PMC6364792

[ejn15839-bib-0029] Ersche, K. D. , Jones, P. S. , Williams, G. B. , Turton, A. J. , Robbins, T. W. , & Bullmore, E. T. (2012). Abnormal brain structure implicated in stimulant drug addiction. Science, 335, 601–604. 10.1126/science.1214463 22301321

[ejn15839-bib-0030] Ersche, K. D. , Meng, C. , Ziauddeen, H. , Stochl, J. , Williams, G. B. , Bullmore, E. T. , & Robbins, T. W. (2020). Brain networks underlying vulnerability and resilience to drug addiction. Proceedings of the National Academy of Sciences of the United States of America, 117, 15253–15261. 10.1073/pnas.2002509117 32541059 PMC7334452

[ejn15839-bib-0031] Ersche, K. D. , Turton, A. J. , Pradhan, S. , Bullmore, E. T. , & Robbins, T. W. (2010). Drug addiction endophenotypes: Impulsive versus sensation‐seeking personality traits. Biological Psychiatry, 68, 770–773. 10.1016/j.biopsych.2010.06.015 20678754 PMC3485555

[ejn15839-bib-0032] Everitt, B. J. (1990). Sexual motivation: A neural and behavioural analysis of the mechanisms underlying appetitive and copulatory responses of male rats. Neuroscience and Biobehavioral Reviews, 14, 217–232. 10.1016/S0149-7634(05)80222-2 2190121

[ejn15839-bib-0033] Everitt, B. J. , Giuliano, C. , & Belin, D. (2018). Addictive behaviour in experimental animals: Prospects for translation. Philosophical Transactions of the Royal Society of London. Series B, Biological Sciences, 373, 20170027. 10.1098/rstb.2017.0027 29352026 PMC5790825

[ejn15839-bib-0034] Ford, J. M. , Morris, S. E. , Hoffman, R. E. , Sommer, I. , Waters, F. , McCarthy‐Jones, S. , Thoma, R. J. , Turner, J. A. , Keedy, S. K. , Badcock, J. C. , & Cuthbert, B. N. (2014). Studying hallucinations within the NIMH RDoC framework. Schizophrenia Bulletin, 40(Suppl 4), S295–S304. 10.1093/schbul/sbu011 24847862 PMC4141312

[ejn15839-bib-0035] Forgy, E. W. (1965). Cluster analysis of multivariate data ‐ efficiency vs interpretability of classifications. Biometrics, 21, 768–769.

[ejn15839-bib-0036] Fouyssac, M. , Pena‐Oliver, Y. , Puaud, M. , Lim, N. T. Y. , Giuliano, C. , Everitt, B. J. , & Belin, D. (2022). Negative urgency exacerbates relapse to cocaine seeking after abstinence. Biological Psychiatry, 91, 1051–1060. 10.1016/j.biopsych.2021.10.009 34922736

[ejn15839-bib-0037] Fouyssac, M. , Puaud, M. , Ducret, E. , Marti‐Prats, L. , Vanhille, N. , Ansquer, S. , Zhang, X. , Belin‐Rauscent, A. , Giuliano, C. , Houeto, J. L. , Everitt, B. J. , & Belin, D. (2021). Environment‐dependent behavioral traits and experiential factors shape addiction vulnerability. The European Journal of Neuroscience, 53, 1794–1808. 10.1111/ejn.15087 33332672

[ejn15839-bib-0038] Fung, G. (2001). A comprehensive overview of basic clustering algorithms. City.

[ejn15839-bib-0039] Giuliano, C. , Marti‐Prats, L. , Domi, A. , Puaud, M. , Pena‐Oliver, Y. , McKenzie, C. , Everitt, B.J. & Belin, D. (2022). The development of compulsive coping behaviours depends on the engagement of dorsolateral dopamine‐dependent mechanisms. BiorXiv. 10.1038/s41380-023-02256-z PMC10914627 37770577

[ejn15839-bib-0040] Giuliano, C. , Puaud, M. , Cardinal, R. N. , Belin, D. , & Everitt, B. J. (2021). Individual differences in the engagement of habitual control over alcohol seeking predict the development of compulsive alcohol seeking and drinking. Addiction Biology, 26, e13041. 10.1111/adb.13041 33955649

[ejn15839-bib-0041] Grant, B. F. , Stinson, F. S. , Dawson, D. A. , Chou, S. P. , Dufour, M. C. , Compton, W. , Pickering, R. P. , & Kaplan, K. (2004). Prevalence and co‐occurrence of substance use disorders and independent mood and anxiety disorders: Results from the National Epidemiologic Survey on alcohol and related conditions. Archives of General Psychiatry, 61, 807–816. 10.1001/archpsyc.61.8.807 15289279

[ejn15839-bib-0042] Grant, B. F. , Stinson, F. S. , & Harford, T. C. (2001). Age at onset of alcohol use and DSM‐IV alcohol abuse and dependence: A 12‐year follow‐up. Journal of Substance Abuse, 13, 493–504. 10.1016/S0899-3289(01)00096-7 11775078

[ejn15839-bib-0043] Harada, M. , Pascoli, V. , Hiver, A. , Flakowski, J. , & Lüscher, C. (2021). Cortico‐striatal activity driving compulsive reward‐seeking. Biological Psychiatry, 90, 808–818. 10.1016/j.biopsych.2021.08.018 34688471

[ejn15839-bib-0044] Herzig, D. A. , Sullivan, S. , Lewis, G. , Corcoran, R. , Drake, R. , Evans, J. , Nutt, D. , & Mohr, C. (2015). Hemispheric language asymmetry in first episode psychosis and schizotypy: The role of cannabis consumption and cognitive disorganization. Schizophrenia Bulletin, 41(Suppl 2), S455–S464. 10.1093/schbul/sbu179 25543118 PMC4373630

[ejn15839-bib-0045] Insel, T. R. (2014). The NIMH research domain criteria (RDoC) project: Precision medicine for psychiatry. The American Journal of Psychiatry, 171, 395–397. 10.1176/appi.ajp.2014.14020138 24687194

[ejn15839-bib-0046] Jadhav, K. S. , Magistretti, P. J. , Halfon, O. , Augsburger, M. , & Boutrel, B. (2017). A preclinical model for identifying rats at risk of alcohol use disorder. Scientific Reports, 7, 9454. 10.1038/s41598-017-09801-1 28842608 PMC5572732

[ejn15839-bib-0047] Jadhav, K. S. , Peterson, V. L. , Halfon, O. , Ahern, G. , Fouhy, F. , Stanton, C. , Dinan, T. G. , Cryan, J. F. , & Boutrel, B. (2018). Gut microbiome correlates with altered striatal dopamine receptor expression in a model of compulsive alcohol seeking. Neuropharmacology, 141, 249–259. 10.1016/j.neuropharm.2018.08.026 30172845

[ejn15839-bib-0048] Kasanetz, F. , Deroche‐Gamonet, V. , Berson, N. , Balado, E. , Lafourcade, M. , Manzoni, O. , & Piazza, P. V. (2010). Transition to addiction is associated with a persistent impairment in synaptic plasticity. Science, 328, 1709–1712. 10.1126/science.1187801 20576893

[ejn15839-bib-0049] Kasanetz, F. , Lafourcade, M. , Deroche‐Gamonet, V. , Revest, J. M. , Berson, N. , Balado, E. , Fiancette, J. F. , Renault, P. , Piazza, P. V. , & Manzoni, O. J. (2013). Prefrontal synaptic markers of cocaine addiction‐like behavior in rats. Molecular Psychiatry, 18, 729–737. 10.1038/mp.2012.59 22584869

[ejn15839-bib-0050] Kumar, R. , & Indrayan, A. (2011). Receiver operating characteristic (ROC) curve for medical researchers. Indian Pediatrics, 48, 277–287. 10.1007/s13312-011-0055-4 21532099

[ejn15839-bib-0051] Kupfer, D. J. , First, M. B. & Regier, D. A. (2008). A research agenda for DSM V.

[ejn15839-bib-0052] Kwako, L. E. , Momenan, R. , Litten, R. Z. , Koob, G. F. , & Goldman, D. (2016). Addictions Neuroclinical assessment: A neuroscience‐based framework for addictive disorders. Biological Psychiatry, 80, 179–189. 10.1016/j.biopsych.2015.10.024 26772405 PMC4870153

[ejn15839-bib-0053] Kwako, L. E. , Schwandt, M. L. , Ramchandani, V. A. , Diazgranados, N. , Koob, G. F. , Volkow, N. D. , Blanco, C. , & Goldman, D. (2019). Neurofunctional domains derived from deep behavioral phenotyping in alcohol use disorder. The American Journal of Psychiatry, 176, 744–753. 10.1176/appi.ajp.2018.18030357 30606047 PMC6609498

[ejn15839-bib-0054] Leggio, L. , Kenna, G. A. , Fenton, M. , Bonenfant, E. , & Swift, R. M. (2009). Typologies of alcohol dependence. From Jellinek to genetics and beyond. Neuropsychology Review, 19, 115–129. 10.1007/s11065-008-9080-z 19184441

[ejn15839-bib-0055] Lubke, G. H. , & Muthen, B. (2005). Investigating population heterogeneity with factor mixture models. Psychological Methods, 10, 21–39. 10.1037/1082-989X.10.1.21 15810867

[ejn15839-bib-0056] Luscher, C. , Robbins, T. W. , & Everitt, B. J. (2020). The transition to compulsion in addiction. Nature Reviews. Neuroscience, 21, 247–263. 10.1038/s41583-020-0289-z 32231315 PMC7610550

[ejn15839-bib-0057] Mann, K. , & Hermann, D. (2010). Individualised treatment in alcohol‐dependent patients. European Archives of Psychiatry and Clinical Neuroscience, 260(Suppl 2), 116–S120. 10.1007/s00406-010-0153-7 20953618

[ejn15839-bib-0058] McDermott, C. , & Kelly, J. P. (2008). Comparison of the behavioural pharmacology of the Lister‐hooded with 2 commonly utilised albino rat strains. Progress in Neuro‐Psychopharmacology & Biological Psychiatry, 32, 1816–1823. 10.1016/j.pnpbp.2008.08.004 18727950

[ejn15839-bib-0059] Morrow, J. D. , & Flagel, S. B. (2016). Neuroscience of resilience and vulnerability for addiction medicine: From genes to behavior. Progress in Brain Research, 223, 3–18. 10.1016/bs.pbr.2015.09.004 26806768

[ejn15839-bib-0060] Murray, J. E. , Belin, D. , & Everitt, B. J. (2012). Double dissociation of the dorsomedial and dorsolateral striatal control over the acquisition and performance of cocaine seeking. Neuropsychopharmacology, 37, 2456–2466. 10.1038/npp.2012.104 22739470 PMC3442340

[ejn15839-bib-0061] Nestler, E. J. (2005). Is there a common molecular pathway for addiction? Nature Neuroscience, 8, 1445–1449. 16251986 10.1038/nn1578

[ejn15839-bib-0062] Pascoli, V. , Hiver, A. , Van Zessen, R. , Loureiro, M. , Achargui, R. , Harada, M. , Flakowski, J. , & Luscher, C. (2018). Stochastic synaptic plasticity underlying compulsion in a model of addiction. Nature, 564, 366–371. 10.1038/s41586-018-0789-4 30568192

[ejn15839-bib-0063] Pascoli, V. , Terrier, J. , Espallergues, J. , Valjent, E. , O'Connor, E. C. , & Lüscher, C. (2014). Contrasting forms of cocaine‐evoked plasticity control components of relapse. Nature, 509, 459–464. 10.1038/nature13257 24848058

[ejn15839-bib-0064] Pedregosa, F. , Varoquaux, G. , Gramfort, A. , Michel, V. , Thirion, B. , Grisel, O. , Blondel, M. , Prettenhofer, P. , Weiss, R. , Dubourg, V. , Vanderplas, J. , Passos, A. , Cournapeau, D. , Brucher, M. , Perrot, M. , & Duchesnay, E. (2011). Scikit‐learn: Machine learning in Python. Journal of Machine Learning Research, 12, 2825–2830.

[ejn15839-bib-0065] Pelloux, Y. , Murray, J. E. , & Everitt, B. J. (2015). Differential vulnerability to the punishment of cocaine related behaviours: Effects of locus of punishment, cocaine taking history and alternative reinforcer availability. Psychopharmacology, 232, 125–134. 10.1007/s00213-014-3648-5 24952093 PMC4281358

[ejn15839-bib-0066] Piazza, P. V. , & Deroche‐Gamonet, V. (2013). A multistep general theory of transition to addiction. Psychopharmacology, 229, 387–413. 10.1007/s00213-013-3224-4 23963530 PMC3767888

[ejn15839-bib-0067] Pohorala, V. , Enkel, T. , Bartsch, D. , Spanagel, R. , & Bernardi, R. E. (2021). Sign‐ and goal‐tracking score does not correlate with addiction‐like behavior following prolonged cocaine self‐administration. Psychopharmacology, 238, 2335–2346. 10.1007/s00213-021-05858-z 33950271 PMC8292273

[ejn15839-bib-0068] Puhl, M. D. , Blum, J. S. , Acosta‐Torres, S. , & Grigson, P. S. (2012). Environmental enrichment protects against the acquisition of cocaine self‐administration in adult male rats, but does not eliminate avoidance of a drug‐associated saccharin cue. Behavioural Pharmacology, 23, 43–53. 10.1097/FBP.0b013e32834eb060 22157144 PMC3650841

[ejn15839-bib-0069] Radwanska, K. , & Kaczmarek, L. (2012). Characterization of an alcohol addiction‐prone phenotype in mice. Addiction Biology, 17, 601–612. 10.1111/j.1369-1600.2011.00394.x 22017485

[ejn15839-bib-0070] Reynolds, D. (2009). Gaussian Mixture Models. In S. Z. Li & A. Jain (Eds.), Encyclopedia of biometrics (pp. 659–663). Springer. 10.1007/978-0-387-73003-5_196

[ejn15839-bib-0071] Swendsen, J. , Conway, K. P. , Degenhardt, L. , Dierker, L. , Glantz, M. , Jin, R. , Merikangas, K. R. , Sampson, N. , & Kessler, R. C. (2009). Socio‐demographic risk factors for alcohol and drug dependence: The 10‐year follow‐up of the national comorbidity survey. Addiction, 104, 1346–1355. 10.1111/j.1360-0443.2009.02622.x 19549055 PMC2794245

[ejn15839-bib-0072] Swendsen, J. , Conway, K. P. , Degenhardt, L. , Glantz, M. , Jin, R. , Merikangas, K. R. , Sampson, N. , & Kessler, R. C. (2010). Mental disorders as risk factors for substance use, abuse and dependence: Results from the 10‐year follow‐up of the National Comorbidity Survey. Addiction, 105, 1117–1128. 10.1111/j.1360-0443.2010.02902.x 20331554 PMC2910819

[ejn15839-bib-0073] Ungless, M. A. , Whistler, J. L. , Malenka, R. C. , & Bonci, A. (2001). Single cocaine exposure in vivo induces long‐term potentiation in dopamine neurons. Nature, 411, 583–587. 10.1038/35079077 11385572

[ejn15839-bib-0074] Witkiewitz, K. , Litten, R. Z. , & Leggio, L. (2019). Advances in the science and treatment of alcohol use disorder. Science Advances, 5, eaax4043. 10.1126/sciadv.aax4043 31579824 PMC6760932

[ejn15839-bib-0075] Woody, M. L. , & Gibb, B. E. (2015). Integrating NIMH research domain criteria (RDoC) into depression research. Current Opinion in Psychology, 4, 6–12. 10.1016/j.copsyc.2015.01.004 25642446 PMC4306327

[ejn15839-bib-0076] Zou, J. , Han, Y. , & So, S. S. (2008). Overview of artificial neural networks. Methods in Molecular Biology, 458, 15–23. 19065803 10.1007/978-1-60327-101-1_2

